# Epidemiological trends, relative survival, and prognosis risk factors of WHO Grade III gliomas: A population‐based study

**DOI:** 10.1002/cam4.2164

**Published:** 2019-04-24

**Authors:** Jun‐Hao Fang, Dong‐Dong Lin, Xiang‐Yang Deng, Dan‐Dong Li, Han‐Song Sheng, Jian Lin, Nu Zhang, Bo Yin

**Affiliations:** ^1^ Department of Neurosurgery The Second Affiliated Hospital and Yuying Children's Hospital of Wenzhou Medical University Wenzhou, Zhejiang China

**Keywords:** anaplastic glioma, incidence, mortality, nomogram, prevalence

## Abstract

**Background:**

Population‐based studies on grade III gliomas are still lacking. The purpose of our study was to investigate epidemiological characteristics, survival, and risk factors of these tumors.

**Patients and methods:**

All data of patients with grade III gliomas were extracted from the Surveillance, Epidemiology, and End Results database. This database provides analysis to evaluate age‐adjusted incidence, incidence‐based mortality, and limited‐duration prevalence. The trends of incidence and mortality were modeled using Joinpoint program. Relative survival was also available in this database. Univariate and multivariate analyses were used to access the prognostic significance of risk factors on cancer‐specific survival. Nomogram was constructed to predict 3‐, 5‐, and 10‐year survival.

**Results:**

Our study showed that during 2000‐2013, the incidence was stable and the mortality rate dropped significantly with APC as −1.95% (95% CI: −3.35% to −0.54%). Patients aged 40‐59 had the highest prevalent cases. The 1‐, 3‐, 5‐, and 10‐year relative survival rates for all patients were 74.7%, 52.8%, 44.4%, and 32.4%. And it varied by risk factors. Cox regression analysis showed older age, male, black race, divorced status, histology of AA, tumor size <3.5 cm and no surgery were associated with worse survival.

**Conclusion:**

Our study provides reasonable estimates of the incidence, mortality, and prevalence for patients with grade III gliomas during 2000‐2013. The results of relative survival and Cox regression analysis revealed that age, race, sex, year of diagnosis, tumor site, histologic type, tumor size, and surgery were the identifiable prognostic indicators. The effects of radiotherapy still need further study. We integrated these risk factors to construct an effective clinical prediction model.

## 1 INTRODUCTION

WHO Grade III gliomas are relatively rare tumor, mainly divided into anaplastic astrocytoma (AA), anaplastic oligodendroglioma (AO) and anaplastic oligoastrocytoma (AOA) according to the 2016 World Health Organization Classification of Tumors of the Central Nervous System.^1^ They only count for 3.1% (AA: 1.7%; AO: 0.5%; AOA: 0.9%) of primary brain and central nervous system tumors.^2^ However, they are feared tumor types, not only because of their poor prognosis, but also because they seriously affect the quality of life. In recent years, an increasing number of studies focused on the management of WHO Grade II and IV gliomas,^3^, ^4^ while there were few reports of WHO Grade III gliomas because of its low incidence and prevalence. Furthermore, WHO Grade III gliomas were always merged with the more common WHO Grade IV gliomas as “high‐grade gliomas”^5^ to be researched and discussed. When we look at high‐grade gliomas as a group, trends, and characteristics of WHO Grade III gliomas will be obscured because glioblastoma occupies a large proportion of this group. Some studies revealed that there were significant differences of prognosis between WHO Grade III and IV gliomas.^6^

To our knowledge, there have been a lack of large‐samples retrospective studies aiming at epidemiology, survival and prognosis of WHO Grade III gliomas. Because of its malignant manifestation and low survival, it is quite necessary to explore trends in incidence and mortality as well as prognostic risk factors of these tumors.

In this study, we conducted a retrospective analysis with the data extracted from the Surveillance, Epidemiology, and End Results (SEER)^7^ population‐based database to research the incidence, mortality, prevalence, survival, and prognosis risk factors of WHO Grade III gliomas. Meanwhile, we established a risk prediction model to predict 3‐, 5‐, and 10‐year survival based on these risk factors.

## 2 MATERIAL AND METHODS

### 2.1 Data sources

SEER is an authoritative source for cancer statistics in the United States. The SEER database provides information on the incidence, mortality, and prevalence of a definite tumor from 1973‐2015. Furthermore, we can obtain the detailed data about demographic, cancer characteristics and treatment of each case, conducting further analysis. In our study, we focused on adult (age ≥ 18 years old) patients with WHO Grade III malignant gliomas (including histologic type ICD‐O‐3:9401/3 anaplastic astrocytoma; 9451/3 anaplastic oligodendroglioma; 9382/3 anaplastic oligoastrocytoma) between January 2000 and December 2013. Patients were divided into 18‐39, 40‐59, and 60 + years groups according to age.

### 2.2 Incidence, mortality, and prevalence

Incidence is a measure of the probability of occurrence of a given disease in a population within a specified period of time, expressed simply as the number of new cases or as a rate per 100 000 persons per year. Age‐adjusted incidence is a weighted average of the age‐specific (crude) incidence, where the weights are the proportions of persons in the corresponding age groups of a standard population, minimizing the effect of a difference in age distributions. The data were obtained from the SEER 18 registries research database, which covers about 28%^7^ population of the US The age‐adjusted incidence was calculated by year of diagnosis and age groups.

Mortality is the number of reported cancer deaths occurring in a specified population during a year, usually expressed as a rate per 100 000 population at risk. Incidence‐based mortality allows a partitioning of mortality by variables (such as year of diagnosis, age at diagnosis, stage of cancer and histology etc) associated with cancer onset. We used the SEER 9 registries research database to calculate the incidence‐based mortality for patient dead during 2000‐2013 and diagnosed during 1973‐2013 because we need to include cases diagnosed at early phase to guarantee maximum number of deaths and avoid misestimation of the incidence‐based mortality. The incidence‐based mortality was calculated by year of death and age groups.

Limited‐duration prevalence represents the proportion of people alive on a certain day who had a diagnosis of the disease within the past several years. We used the SEER 9 registries research database to calculate the limited‐duration prevalence counts at 1 January 2013 for patients diagnosed from 1975 through 2013. It was calculated by histology groups and age groups.

### 2.3 Relative survival

In survival analysis, relative survival of a cancer was calculated by dividing the overall survival after diagnosis by the survival as observed in a similar population not diagnosed with that cancer. The survival data for WHO grade III glioma were obtained from the SEER 18 registries research database, which contains more population of the US than the SEER 9 registries research database. We estimated 1‐, 3‐, 5‐, and 10‐year relative survival rates for all patients by all variables.

### 2.4 Prognosis research

We extracted the detailed information of each cases from the SEER 18 registries research database to conduct prognosis analysis. The baseline demographics of patients (age, sex, race, year of diagnosis), characteristics of tumor (site, histologic, size), and treatment (radiotherapy, surgery type) were adopted into analysis. We included patients who had only one primary tumor and excluded these whose treatment information (radiotherapy or surgery type) was unknown. The end point of our analysis was cancer‐specific survival according to special code provided by SEER, defined as the interval from diagnosis to death as a result of cancer.

### 2.5 Statistical analysis

Incidence, mortality, prevalence, and relative survival were estimated using SEER*Stat 8.3.5. Incidence and mortality rates were age‐adjusted to the 2000 US standard population. Joinpoint Regression Program 4.6.0.0 was used to evaluate the trends for incidence and mortality. The program also fitted the simplest Joinpoint model that the data allow as well as calculated annual percentage change (APC) and 95% CI. It tested the significance of each APC using a Monte Carlo Permutation method.^8^ Descriptive analysis was used to count the distribution of patients according to variables. Univariate and multivariate Cox proportional hazards models were used to analyze the influence of each variable with survival and expressed as hazard ratios (HR) and 95% confidence intervals (CI).

A nomogram was established to predict 3‐, ‐, and 10‐year survival based on the result of multivariate Cox analysis. We achieve it using the package of rms^9^ in R version 3.5.0. Concordance index (C‐index) and calibration curve were used to assess the performance of the established nomogram model.

A two‐sided *P* value of 0.05 was considered statistically significant. Descriptive analysis and Cox proportional hazards models were executed with SPSS 25.0 and all figures were depicted by GraphPad Prism 7.0 or R version 3.5.0.

## 3 RESULTS

### 3.1 Incidence, mortality, and prevalence

**Figure 1:**
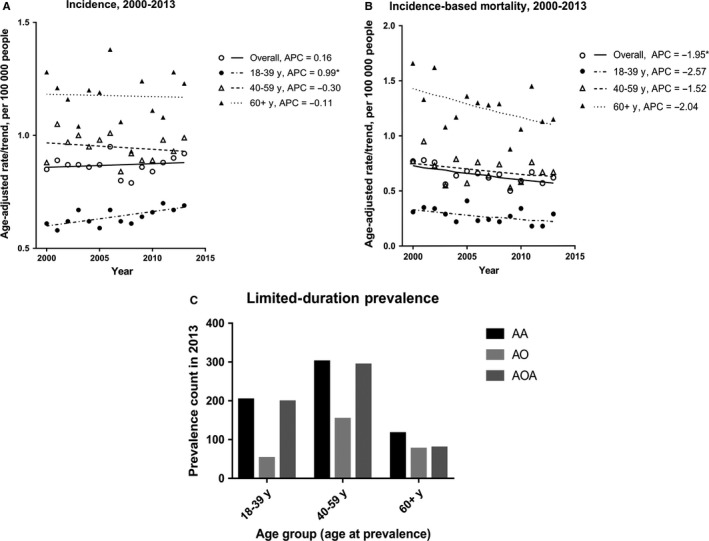
Incidence, mortality, and prevalence of WHO Grade III gliomas during 2000‐2013. (A) Trends in annual grade III gliomas age‐adjusted incidence by age group; (B) Trends in annual grade III gliomas incidence‐based mortality by age group; (C) Limited‐duration prevalence of grade III gliomas by age group and histologic type

The age‐adjusted incidence and incidence‐based mortality of WHO grade III gliomas during 2000‐2013 were described as rates (95% CI) in Table 1, with Joinpoint analysis estimating the trends of them. The overall age‐adjusted incidence rate was 0.87 (95% CI: 0.85 to 0.89) per 100 000 person‐years. The incidence rates increased with age, as 0.64 (95% CI: 0.62‐0.67) in person aged 18‐39 years, 0.95 (95% CI: 0.91‐0.98) in person aged 40‐59 years and 1.17 (95% CI: 1.12‐1.22) in person aged 60 + years. In 2000‐2013, the overall incidence rates had a slight increase with APC as 0.16% (95% CI: −0.55%‐0.87%), but it had no statistical significance. However, the incidence rates increased significantly for 18‐39 years group with APC as 0.99% (95% CI: 0.34%‐1.63%). Nonsignificant decreases were observed for 40‐59 years group (APC = −0.30%; 95% CI: −1.21%‐0.62%) and 60 + years group (APC = −0.11%; 95% CI: −1.57%‐1.38%) between 2000 and 2013 (Figure 1A and Table 1).

**Table 1:** Rates and trends in WHO Grade III gliomas incidence and mortality during 2000‐2013

Age group	Incidence rate (2000‐2013)	Trend			Mortality rate (2000‐2013)	Trend		
		APC	95% CI	*P* value		APC	95% CI	*P* value
Overall	0.87 (0.85, 0.89)	0.16	−0.55, 0.87	0.638	0.64 (0.61, 0.67)	−1.95	−3.35, −0.54	0.011
18‐39	0.64 (0.62, 0.67)	0.99	0.34, 1.63	0.006	0.28 (0.25, 0.31)	−2.57	−5.65, 0.61	0.103
40‐59	0.95 (0.91, 0.98)	−0.30	−1.21, 0.62	0.489	0.69 (0.64, 0.74)	−1.52	−3.66, 0.67	0.154
60+	1.17 (1.12, 1.22)	−0.11	−1.57, 1.38	0.876	1.25 (1.17, 1.34)	−2.04	−4.07, 0.03	0.053

Abbreviation: APC, annual percentage change.

The incidence‐based mortality of WHO grade III gliomas overall was 0.64 (95% CI: 0.61‐0.67) per 100 000 person‐years. (Table 1) For different age groups, the incidence‐based mortality also increased with age, from 0.28 (95% CI: 0.25‐0.31) for 18‐39 years group to 1.25 (95% CI: 1.17‐1.34) for 60 + years group (Table 1). The trend of overall mortality rates was significant, with a −1.95% (95% CI: −3.35%‐0.54%) decline per year during 2000‐2013. But, the trends of mortality rates for each age group demonstrated no significant decline, −2.57% (95% CI: −5.65%‐0.61%) for patients aged 18‐39 years, −1.52% (95% CI: −3.66%‐0.67%) for patients aged 40‐59 years and −2.04% (95% CI: −4.07%‐0.03%) for patients aged 60 + years (Figure 1B and Table 1).

The prevalent distribution of various histological subtypes in 2013 varied greatly by age groups (Figure 1C). Patients aged 40‐59 years had the highest prevalent cases than the other two groups (40‐59 years: N = 747; 18‐39 years: N = 453; 60 + years: N = 271). By contrast, AO had fewer prevalent cases, especially in the groups of 18‐39 years and 40‐59 years. The prevalent number of AA was almost the same as AOA for patients aged 18‐39 years or 40‐59 years, while AA was more prevalent for patients aged 60 + years.

Number of cases or deaths and age‐adjusted incidence and incidence‐based mortality per year are shown in Table S1.

### 3.2 Survival

**Figure 2:**
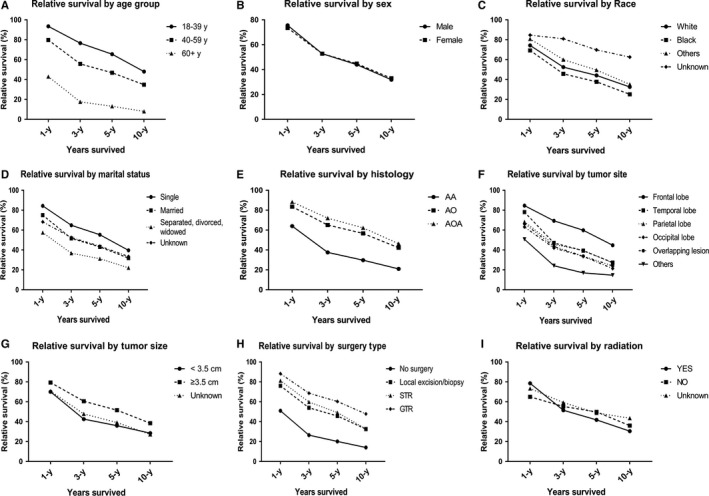
Relative survival of WHO Grade III gliomas by different variables. (A) Age group; (B) Sex; (C) Race; (D) Marital status; (E) Histology; (F) Tumor site; (G) Tumor size; (H) Surgery type; (I) Radiation

We included a total of 6,720 patients for survival analysis (Table S2 and Figure 2). The overall 1‐, 3‐, 5‐, and 10‐year relative survival rates (RSR) were 74.7%, 52.8%, 44.4%, and 32.4%. Relative survival varied by different variables except sex. For age groups, relative survival rates declined with increasing age (5‐year RSR: 65.5% [95% CI: 63.1%‐67.7%] for 18‐39 years; 46.9% [95% CI: 44.7%‐49.0%] for 40‐59 years; 13.2% [95% CI: 11.3%‐15.2%] for 60 + years). Other races had higher relative survival than Whites, while the RSRs of Blacks were the lowest. For marital status, singles had better relative survival than married person, while separated, divorced, or widowed person had a worst survival. Focus on histology, the relative survival rates of AA were remarkably lower than AO and AOA (such as 5‐year RSR for AA: 29.8% [95% CI: 28.0%‐31.5%]; 5‐year RSR for AO:56.7% [95% CI: 53.5%‐59.8%]; 5‐year RSR for AOA: 62.3% [95% CI: 59.7%‐64.7%]). By contrast, tumors occurred in the frontal lobe had higher relative survival than occurred in other sites. Interestingly, the RSRs of tumor size ≥ 3.5cm (5‐year RSR: 51.6% [95% CI: 49.6%‐53.6%]) was higher than tumor size < 3.5 cm (5‐year RSR: 36% [95% CI: 33.0%‐39.0%]). Patients who received GTR had better survival than those who received STR or biopsy, as patients without surgery had worst survival. The 1‐year RSR of patients received radiation was higher than patients who did not receive radiation, while its 3‐, 5‐, and 10‐year RSR were lower than no radiation group.

### 3.3 Univariate and multivariate analysis

After removing some cases whose treatment information was unknow, we finally included 5907 patients for univariate and multivariate analysis (Table 2). A Cox proportional hazards model was used to identify the independent prognostic factors for cancer‐specific survival with *P* < 0.05. Using 18‐39 years group as the reference, mortality increased with increasing age (40‐59 years: HR: 1.674, 95% CI: 1.513‐1.852; 60 + years: HR: 4.354, 95% CI: 3.906‐4.854; *P* < 0.001). The black race was associated with worse CSS as compared to the white race (HR: 1.224, 95% CI: 1.053‐1.422, *P* = 0.008). Female had better CSS than male (HR: 0.879, 95% CI: 0.816‐0.946, *P* = 0.001), although there was no statistical significance in univariate analysis (*P* = 0.428). Tumor histology with AO (HR: 0.610, 95% CI: 0.547‐0.679, *P* < 0.001) or AOA (HR: 0.535, 95% CI: 0.486‐0.590, *P* < 0.001) had a significantly decreased mortality as compare to AA. Tumor size ≥ 3.5 cm had a better CSS than tumor size < 3.5 cm (HR: 0.869, 95% CI: 0.789‐0.957, *P* < 0.001). After surgery, patients had a significant better CSS compared to no surgery. No radiation treatment was associated with higher mortality (HR: 1.089, 95% CI: 0.995‐1.193, *P* = 0.065), but it was not statistically significant. The result of multivariate analysis revealed that other independent prognostic factors were year of diagnosis, marital status, and tumor site.

**Table 2:** Univariate and multivariable analysis of cancer‐specific survival

Variables	Total (%) (n = 5907)	Univariate analysis			Multivariable analysis		
		HR	95% CI	*P* value	HR	95% CI	*P* value
Age group							0.000
18‐39 years	1966 (33.3)	Ref	‐	‐	Ref	‐	‐
40‐59 years	2439 (41.3)	1.776	1.614, 1.955	0.000	1.674	1.513, 1.852	0.000
≥60 years	1502 (25.4)	5.783	5.237, 6.386	0.000	4.354	3.906, 4.854	0.000
Race							0.004
White	5131 (86.9)	Ref	‐	‐	Ref	‐	‐
Black	322 (5.5)	1.209	1.042, 1.402	0.012	1.224	1.053, 1.422	0.008
Others	423 (7.2)	0.775	0.666, 0.903	0.001	0.841	0.721, 0.979	0.026
Unknown	31 (0.5)	0.662	0.330, 1.324	0.243	0.726	0.362, 1.457	0.368
Sex							
Male	3323 (56.3)	Ref	‐	‐	Ref	‐	‐
Female	2584 (43.7)	0.971	0.903, 1.044	0.428	0.879	0.816, 0.946	0.001
Year of diagnosis							0.000
2000‐2004	2002 (33.9)	Ref	‐	‐	Ref	‐	‐
2005‐2009	2078 (35.2)	0.820	0.756, 0.889	0.000	0.819	0.754, 0.889	0.000
2010‐2013	1827 (30.9)	0.736	0.663, 0.817	0.000	0.705	0.633, 0.784	0.000
Marital status							0.008
Single	1390 (23.5)	Ref	‐	‐	Ref	‐	‐
Married	3567 (60.4)	1.483	1.348, 1.632	0.000	1.042	0.941, 1.153	0.430
Separated, divorced, widowed	757 (12.8)	2.111	1.866, 2.389	0.000	1.230	1.079, 1.403	0.002
Unknown	193 (3.3)	1.434	1.151, 1.787	0.001	1.089	0.873, 1.359	0.450
Tumor site							0.000
Frontal lobe	2477 (41.9)	Ref	‐	‐	Ref	‐	‐
Temporal lobe	1199 (20.3)	1.802	1.630, 1.993	0.000	1.446	1.305, 1.601	0.000
Parietal lobe	688 (11.6)	1.873	1.663, 2.110	0.000	1.456	1.290, 1.642	0.000
Occipital lobe	118 (2.0)	2.348	1.853, 2.976	0.000	1.662	1.309, 2.111	0.000
Overlapping lesion of brain	716 (12.1)	2.043	1.823, 2.290	0.000	1.466	1.304, 1.649	0.000
Others	709 (12.0)	3.500	3.136, 3.907	0.000	2.150	1.914, 2.416	0.000
Histologic type							0.000
AA	3063 (51.9)	Ref	‐	‐	Ref	‐	‐
AO	1095 (18.5)	0.475	0.429, 0.526	0.000	0.610	0.547, 0.679	0.000
AOA	1749 (29.6)	0.394	0.359, 0.431	0.000	0.535	0.486, 0.590	0.000
Tumor size							0.013
<3.5 cm	1219 (20.6)	Ref	‐	‐	Ref	‐	‐
≥3.5 cm	2839 (48.1)	0.657	0.598, 0.721	0.000	0.869	0.789, 0.957	0.000
Unknown	1849 (31.3)	0.912	0.828, 1.005	0.063	0.891	0.807, 0.983	0.021
Radiotherapy							
Yes	4368 (73.9)	Ref	‐	‐	Ref	‐	‐
No	1539 (26.1)	0.814	0.747, 0.887	0.000	1.089	0.995, 1.193	0.065
Surgery							0.000
No surgery	1456 (24.6)	Ref	‐	‐	Ref	‐	‐
Local excision/biopsy	1110 (18.8)	0.441	0.398, 0.490	0.000	0.631	0.566, 0.702	0.000
STR	1587 (26.9)	0.403	0.367, 0.443	0.000	0.650	0.587, 0.718	0.000
GTR	1745 (29.7)	0.287	0.260, 0.317	0.000	0.505	0.454, 0.562	0.000

Abbreviations: AA, anaplastic astrocytoma; AO, anaplastic oligodendroglioma; AOA, anaplastic oligoastrocytoma; GTR, Gross total resection; HR, hazard ratio; STR, Subtotal resection.

**Figure 3:**
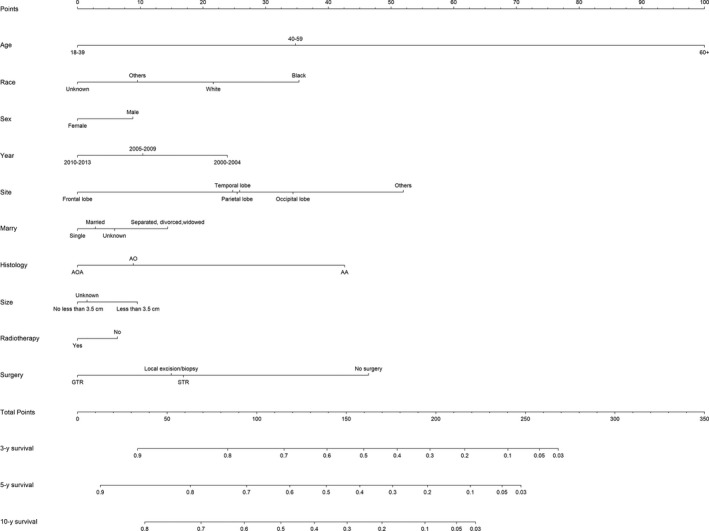
A cancer‐specific survival nomogram for patients with WHO Grade III gliomas. An individual patient's value is located on each variable axis, and a line is drawn upward to determine the number of points received for each variable value. The sum of these numbers is located on the Total Points axis, and a line is drawn downward to the survival axes to determine the likelihood of 3‐, 5‐, or 10‐year survival

**Figure 4:**
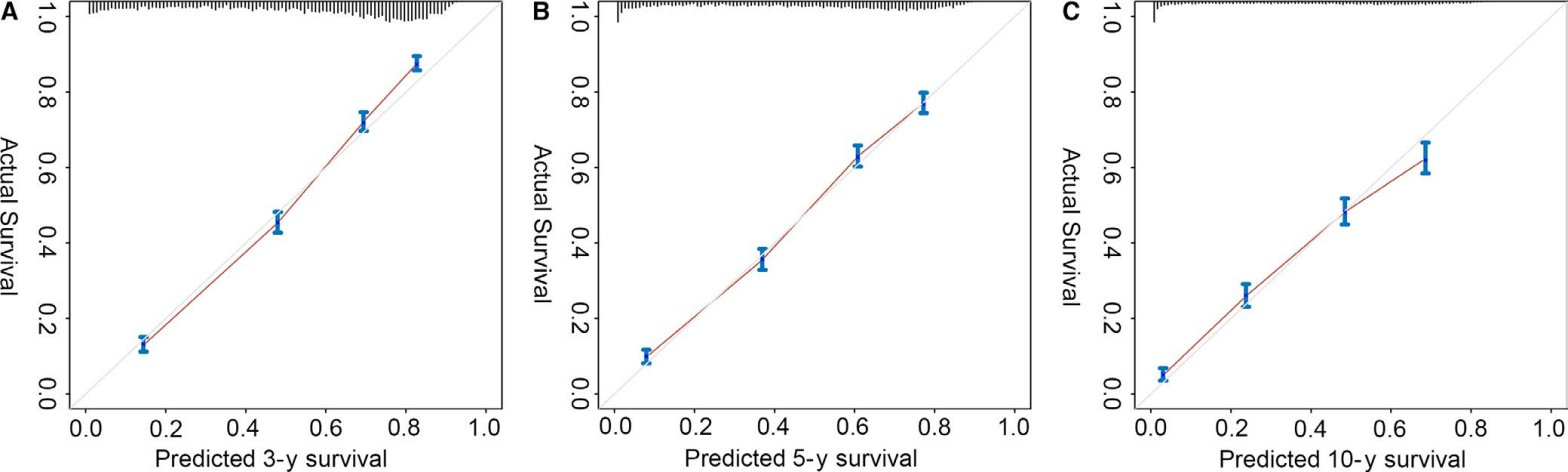
The calibration curve of the nomogram. Nomogram‐predicted survival is plotted on the x‐axis; actual survival is plotted on the y‐axis. Check up the accuracy of the nomogram at (A) 3‐year, (B) 5‐year, and (C) 10‐year survival in the primary cohort

Then, we established a nomogram (Figure 3) to predict 3‐, 5‐, and 10‐year survival intuitively based on variables of multivariate Cox analysis. We selected all independent factors into the nomogram. The C‐index was 0.763 (95% CI: 0.755‐0.771). What is more, the survival probability calibration curve (Figure 4) were in good agreement between prediction by nomogram and actual observation.

## 4 DISCUSSION

We conducted a retrospective study to analyze epidemiological trend, relative survival, and prognostic risk factors for patients diagnosed as AA, AO, and AOA between 2000 and 2013. SEER database covers cases from 1973.^7^ But we culled the data before 2000 for several reasons. One important reason is that the classification of WHO Grade III gliomas was inaccurate in the 1970s, 1980s, and 1990s, leading to a significant difference in epidemiological data with 2000‐2013. Another reason is that the criteria for diagnosis and treatment may differ greatly between registries before 2000, which might cause significant bias. We believe that the period from 2000 to 2013 is appropriate for analysis, because the classification and treatment of WHO Grade III gliomas were relatively homogeneous during the period.

Consistent with previous studies,^10^ we found that the incidence rises with age, which may indicate a long‐term accumulation of risk factors plays an important role in the cause of WHO Grade III gliomas.^11^ We also found that the incidence trends were stable for overall groups, 40‐59 years group and 60 + years group, except for 18‐39 years group with a significantly increased incidence (APC = 0.99%). Hess, KR et al^10^ showed an significant increase in the incidence of AA, AO, AOA between 1977 and 2000 using SEER data. It is not incompatible with our results. A number of studies have attributed the increases to advances in diagnostic equipment and biopsy technique, especially the introduction of CT in 1970s and MRI in 1980s.^12^, ^13^, ^14^ But the effect of this aspect was weak in the 21st century because of the widespread use of CT, MRI and stereotactic brain biopsy procedure. It is a reasonable assumption that the increased incidence of 18‐39 years group was mainly due to internal and environmental risk factors, such as ionizing radiation, cell phones, foods containing N‐nitroso compounds, and etc^15^, ^16^, ^17^ Mortality analysis showed that after 2000, the mortality rate was significantly decreasing with APC as −1.95% for all population. The decreasing mortality rate among patients with WHO Grade III gliomas suggest that we have achieved certain effect in the management of this tumor. However, research on grade III gliomas is still lacking, and clinical trials to establish treatment standards are in progress. What is more, prevalence study revealed 40‐59 years group had the most patients diagnosed as WHO Grade III gliomas comparing with other two age groups. Patients aged 40‐59 years may be a group worthy of attention, and cause a great social burden.

Over the past few decades, a number of studies have been conducted to analyze risk factors associated with survival among patients diagnosed as grade III gliomas. Patient age, KPS, histologic type, and extent of surgical resection are generally accepted factors influencing the prognosis at present.^18^, ^19^, ^20^ And we also investigated some uncommon factors such as year of diagnosis, marital status, tumor site, radiotherapy, etc For each risk factor, we comprehensively evaluated the results of relative survival analysis and Cox regression analysis to obtain accurate conclusions. In our study, we found age was the most significant prognostic factor as the risk of mortality for patients aged 60 + years was 4.354 folds than patients aged 18‐39 years. Gorlia et al^21^ also showed that younger age had a significant positive prognostic value for progression free survival and overall survival of AO and AOA. We also found that patients with frontal location had a significantly better prognosis than those with tumor in other locations, which is consistent with the findings of Gorlia et al^21^ Interestingly, our data showed that tumor size ≥ 3.5 cm had a better survival than tumor size < 3.5 cm and multivariate analysis also supported this result, which is contrary to previous studies. A reasonable explanation is that patients with larger tumors might accept more aggressive treatments and achieved a good impact on prognosis.

With the update of the WHO classification for CNS tumor in 2016, the diagnosis of gliomas shifted from histology to genetics, especially 1p/19q status and IDH mutations.^22^, ^23^, ^24^ A large number of studies have focused on the treatment options for grade III glioma with different molecular markers.^25^, ^26^ However, the latest NCCN guidelines for anaplastic gliomas recommend early and maximal surgical resection as the first therapeutic option.^27^ Our result also showed that surgical removal of tumors can improve prognosis and GTR of tumors had a best survival. But, there are currently no RCTs on the benefits of extensive surgery. Another study by Fujii Y et al revealed that in patients with AA and AOA, resection of 53% or more of the preoperative T2‐weighted high‐signal intensity volume can achieve a significant survival advantage.^28^ After surgery, radiotherapy with 33 fractions of 1.8 Gy has been the standard of care for anaplastic gliomas. Randomized controlled trials on the dose and efficacy of radiotherapy for anaplastic gliomas have been still lacking. In our study, we found that patients who received radiotherapy had a higher 1‐year relative survival, but they would have a poorer long‐term survival. We suggest that the decision of radiotherapy has to be made by taking into potential adverse effects and benefits. Some studies have shown radiotherapy may cause additional long‐term cognitive disability and a higher symptom burden.^29^ Several trials showed adding chemotherapy to radiotherapy improved outcome for anaplastic gliomas.^25^, ^26^, ^30^ Considering the damage of radiation and chemotherapy on patients, a strategy of “wait and see” has been adopted for patients with low grade gliomas who have had an extensive resection. This strategy is sometimes elected for high‐risk patients who have had surgery, but the risk is correspondingly increased.

There is currently no clinical prediction model based on survival for grade III gliomas. The nomogram provided a visual representation of various risk factors and quantified their relative impact on survival. Our large sample size, relatively perfect risk factor data, homogeneous patient population, and long follow‐up time allowed us to build a well predictive risk model. One major drawback of the nomogram is the absence of external validation because we lacked external data. However, the good C‐index and calibration curve of internal validation still support the accuracy of the prediction model. We hope that the nomogram can effectively promote communication between doctors and patients as well as help them choose more beneficial treatment options.

We must admit some limitations in our study. There are multiple biases that are difficult to solve because it is a retrospective study. Although most of our analysis used data after 2000, we had to include earlier data in order to get accurate results when we analyzed mortality and prevalence. Because SEER database is a system that is constantly improved and updated, the classification and treatment of grade III gliomas were also changing, especially for AOA. It inevitably led to inaccurate results. Another limitation is the lack of information on molecular markers (IDH1/2, 1p/19q co‐deletion status etc) and chemoradiotherapy regimens, which limits our further analysis. We hope that future high‐quality studies on grade III gliomas will confirm our finding.

## 5 CONCLUSION

We have described epidemiological trends, relative survival and risk factors of patients with WHO Grade III gliomas using a large population data. We found the incidence was stable and the mortality rate declined significantly during 2000‐2013. In addition, the results revealed that older age, black race, male, divorced status, non‐frontal location, histology of AA, smaller tumor size, and no surgical resection were associated with worse survival. And the effects of radiotherapy still need further study. Finally, we integrated these risk factors to construct a nomogram to predict 3‐, 5‐, and 10‐year survival.

## DISCLOSURE

The authors report no conflicts of interest in this work.
