# Supplementary material for: Epidemiological trends, relative survival, and prognosis risk factors of WHO Grade III gliomas: A population‐based study
Source: Cancer Med. 2019 Apr 24;8(6):3286–95. doi: 10.1002/cam4.2164 (PMC6558496; doi:10.1002/cam4.2164)
Supplement: Supplementary file 1 [file CAM4-8-3286-s001.docx]

| Table S1. WHO Grade III gliomas incidence and mortality during 2000-2013 according to age groups | | | | | | | | |
| --- | --- | --- | --- | --- | --- | --- | --- | --- |
| Age-adjusted incidence  (SEER-18) | Age at diagnosis | | | | | | | |
| Year of diagnosis | Overall | | 18-39 | | 40-59 | | 60+ | |
|  | No. cases | Rate | No. cases | Rate | No. cases | Rate | No. cases | Rate |
| 2000 | 488 | 0.854 | 157 | 0.608 | 180 | 0.880 | 151 | 1.280 |
| 2001 | 511 | 0.885 | 149 | 0.576 | 218 | 1.047 | 144 | 1.208 |
| 2002 | 511 | 0.866 | 161 | 0.624 | 210 | 0.969 | 140 | 1.158 |
| 2003 | 524 | 0.870 | 174 | 0.671 | 221 | 0.996 | 129 | 1.045 |
| 2004 | 523 | 0.865 | 158 | 0.618 | 213 | 0.948 | 152 | 1.196 |
| 2005 | 513 | 0.867 | 147 | 0.595 | 220 | 0.984 | 146 | 1.192 |
| 2006 | 584 | 0.951 | 172 | 0.671 | 234 | 1.012 | 178 | 1.381 |
| 2007 | 493 | 0.795 | 154 | 0.617 | 195 | 0.836 | 144 | 1.064 |
| 2008 | 503 | 0.794 | 156 | 0.614 | 219 | 0.922 | 128 | 0.928 |
| 2009 | 552 | 0.864 | 163 | 0.642 | 211 | 0.891 | 178 | 1.239 |
| 2010 | 544 | 0.843 | 171 | 0.663 | 212 | 0.888 | 161 | 1.110 |
| 2011 | 584 | 0.884 | 178 | 0.703 | 239 | 0.977 | 167 | 1.076 |
| 2012 | 606 | 0.900 | 178 | 0.674 | 224 | 0.927 | 204 | 1.283 |
| 2013 | 618 | 0.915 | 182 | 0.687 | 241 | 0.987 | 195 | 1.232 |
| Incidence-based mortality  (SEER-9) | Age at death | | | | | | | |
| Year of death | Overall | | 18-39 | | 40-59 | | 60+ | |
|  | No. cases | Rate | No. cases | Rate | No. cases | Rate | No. cases | Rate |
| 2000 | 149 | 0.774 | 27 | 0.307 | 55 | 0.769 | 67 | 1.663 |
| 2001 | 156 | 0.783 | 31 | 0.355 | 70 | 0.947 | 55 | 1.330 |
| 2002 | 151 | 0.762 | 29 | 0.336 | 55 | 0.730 | 67 | 1.616 |
| 2003 | 112 | 0.562 | 25 | 0.294 | 42 | 0.552 | 45 | 1.083 |
| 2004 | 134 | 0.635 | 19 | 0.218 | 63 | 0.792 | 52 | 1.170 |
| 2005 | 139 | 0.680 | 34 | 0.407 | 46 | 0.573 | 59 | 1.364 |
| 2006 | 138 | 0.656 | 19 | 0.229 | 62 | 0.755 | 57 | 1.303 |
| 2007 | 130 | 0.617 | 20 | 0.242 | 51 | 0.644 | 59 | 1.283 |
| 2008 | 140 | 0.645 | 19 | 0.224 | 60 | 0.741 | 61 | 1.286 |
| 2009 | 111 | 0.497 | 23 | 0.268 | 44 | 0.529 | 44 | 0.876 |
| 2010 | 131 | 0.585 | 28 | 0.336 | 51 | 0.584 | 52 | 1.058 |
| 2011 | 150 | 0.669 | 15 | 0.179 | 64 | 0.761 | 71 | 1.448 |
| 2012 | 131 | 0.569 | 16 | 0.182 | 56 | 0.673 | 59 | 1.131 |
| 2013 | 144 | 0.617 | 25 | 0.289 | 56 | 0.668 | 63 | 1.153 |

**Note:** Rates were calculated as number of cases per 100000 person-years and age-adjusted to the 2000 U.S. standard population.

| Table S2. Relative survival of WHO Grade III gliomas | | | | | |
| --- | --- | --- | --- | --- | --- |
| Variables | n | 1-year | 3-year | 5-year | 10-year |
| Overall | 6270 | 74.7% (73.6-75.7%) | 52.8% (51.5-54.1%) | 44.4% (43.0-45.7%) | 32.4% (30.9-34.0%) |
| Age group |  |  |  |  |  |
| 18-39 yrs | 2186 | 93.5% (92.3-94.5%) | 76.6% (74.6-78.5%) | 65.5% (63.1-67.7%) | 48.0% (45.0-51.0%) |
| 40-59 yrs | 2771 | 79.8% (78.2-81.3%) | 55.8% (53.7-57.8%) | 46.9% (44.7-49.0%) | 34.9% (32.4-37.4%) |
| ≥60 yrs | 1763 | 43.0% (40.6-45.4%) | 17.6% (15.6-19.7%) | 13.2% (11.3-15.2%) | 8.0% (6.0-10.3%) |
| Race |  |  |  |  |  |
| White | 5833 | 74.4% (73.2-75.6%) | 52.5% (51.1-53.9%) | 44.2% (42.7-45.7%) | 32.6% (30.9-34.3%) |
| Black | 366 | 69.3% (64.2-73.9%) | 45.8% (40.2-51.2%) | 37.8% (32.2-43.4%) | 25.2% (19.1-31.7%) |
| Others | 484 | 80.9% (77.0-84.3%) | 60.0% (55.1-64.6%) | 49.6% (44.2-54.7%) | 35.2% (28.9-41.5%) |
| Unknown | 37 | 84.7% (66.0-93.6%) | 81.0% (60.8-91.4%) | 69.9% (44.9-85.2%) | 62.6% (35.7-80.9%) |
| Sex |  |  |  |  |  |
| Male | 3769 | 75.6% (74.1-77.0%) | 52.8% (51.0-54.5%) | 44.0% (42.2-45.8%) | 31.9% (29.8-34.0%) |
| Female | 2951 | 73.5% (71.8-75.1%) | 52.8% (50.8-54.8%) | 44.8% (42.8-46.9%) | 33.1% (30.8-35.5%) |
| Marital status |  |  |  |  |  |
| Single | 1584 | 84.4% (82.5-86.2%) | 64.9% (62.3-67.5%) | 55.4% (52.5-58.2%) | 39.7% (36.1-43.2%) |
| Married | 4005 | 75.0% (73.6-76.4%) | 51.7% (50.0-53.4%) | 43.1% (41.3-44.8%) | 31.9% (29.9-33.9%) |
| Separated, divorced, widowed | 883 | 57.5% (54.0-60.8%) | 36.8% (33.4-40.3%) | 31.3% (27.9-34.7%) | 22.1% (18.4-26.0%) |
| Unknown | 248 | 68.3% (61.8-73.9%) | 52.5% (45.5-59.1%) | 43.9% (36.7-50.9%) | 33.5% (25.3-41.8%) |
| Tumor site |  |  |  |  |  |
| Frontal lobe | 2798 | 84.6% (83.1-85.9%) | 69.4% (67.5-71.3%) | 59.9% (57.8-62.0%) | 44.8% (42.1-47.5%) |
| Temporal lobe | 1311 | 78.1% (75.7-80.4%) | 47.1% (44.1-50.1%) | 39.4% (36.3-42.4%) | 27.4% (23.9-31.0%) |
| Parietal lobe | 774 | 68.4% (64.8-71.6%) | 45.6% (41.7-49.4%) | 39.6% (35.6-43.5%) | 27.1% (22.6-31.7%) |
| Occipital lobe | 126 | 63.2% (53.7-71.3%) | 42.0% (32.4-51.3%) | 33.6% (24.3-43.2%) | 21.4% (12.5-32.0%) |
| Overlapping lesion of brain | 831 | 66.5% (63.1-69.7%) | 43.5% (39.8-47.0%) | 33.9% (30.3-37.5%) | 24.0% (20.3-27.9%) |
| Others | 823 | 50.8% (47.2-54.3%) | 24.2% (21.0-27.5%) | 17.0% (14.1-20.2%) | 14.7% (11.7-18.1%) |
| Histologic type |  |  |  |  |  |
| AA | 3528 | 64.0% (62.4-65.7%) | 37.6% (35.8-39.4%) | 29.8% (28.0-31.5%) | 21.0% (19.1-22.9%) |
| AO | 1249 | 83.5% (81.2-85.5%) | 65.1% (62.1-67.8%) | 56.7% (53.5-59.8%) | 42.3% (38.6-46.0%) |
| AOA | 1943 | 88.3% (86.7-89.7%) | 72.0% (69.8-74.2%) | 62.3% (59.7-64.7%) | 46.4% (43.0-49.6%) |
| Tumor size |  |  |  |  |  |
| < 3.5cm | 1362 | 70.1% (67.5-72.6%) | 42.6% (39.8-45.4%) | 36.0% (33.0-39.0%) | 28.5% (25.2-31.9%) |
| ≥3.5cm | 3166 | 79.4% (77.9-80.8%) | 60.6% (58.7-62.4%) | 51.6% (49.6-53.6%) | 38.5% (36.0-40.9%) |
| Unknown | 2175 | 70.7% (68.7-72.7%) | 47.9% (45.6-50.1%) | 39.2% (36.9-41.5%) | 27.2% (24.7-29.7%) |
| Radiotherapy |  |  |  |  |  |
| Yes | 4655 | 78.6% (77.4-79.8%) | 51.5% (49.9-53.0%) | 41.9% (40.3-43.5%) | 30.5% (28.6-32.4%) |
| No | 1903 | 65.0% (62.7-67.1%) | 55.4% (53.0-57.8%) | 49.9% (47.3-52.3%) | 36.1% (33.1-39.0%) |
| Unknown | 162 | 73.4% (65.6-79.8%) | 59.3% (50.6-67.0%) | 48.9% (39.7-57.5%) | 43.5% (33.6-53.0%) |
| Surgery |  |  |  |  |  |
| No surgery | 1688 | 51.0% (48.5-53.4%) | 26.5% (24.3-28.8%) | 20.2% (18.1-22.4%) | 14.1% (11.9-16.4%) |
| Local excision/biopsy | 1256 | 76.1% (73.6-78.5%) | 54% (51.0-57.0%) | 45.8% (42.6-48.9%) | 32.6% (28.8-36.4%) |
| STR | 1773 | 81.3% (79.3-83.1%) | 59.9% (57.3-62.4%) | 49.2% (46.4-51.9%) | 32.9% (29.6-36.2%) |
| GTR | 1908 | 88.3% (86.7-89.7%) | 68.7% (66.3-70.9%) | 60.4% (57.8-62.9%) | 47.9% (44.7-51.0%) |

**Abbreviations:** AA: anaplastic astrocytoma; AO: anaplastic oligodendroglioma; AOA: anaplastic oligoastrocytoma; STR: Subtotal resection; GTR: Gross total resection
